# Mechanical ventilation during extracorporeal membrane oxygenation

**DOI:** 10.1186/cc13702

**Published:** 2014-01-21

**Authors:** Matthieu Schmidt, Vincent Pellegrino, Alain Combes, Carlos Scheinkestel, D Jamie Cooper, Carol Hodgson

**Affiliations:** 1 The Australian & New Zealand Intensive Care Research Centre, Department of Epidemiology and Preventive Medicine, School of Public Health and Preventive Medicine, Level 6, The Alfred Centre, Commercial Road, Melbourne, Victoria 3004, Australia; 2 Intensive Care Department, Alfred Hospital, Commercial Road, Melbourne, Victoria 3004, Australia; 3 Medical-Surgical Intensive Care Unit, iCAN, Institute of Cardiometabolism and Nutrition, Hôpital de la Pitié – Salpêtrière, Assistance Publique – Hôpitaux de Paris, 47 boulevard de l’hôpital, 75651, Paris, Cedex 13, France

## Abstract

The timing of extracorporeal membrane oxygenation (ECMO) initiation and its outcome in the management of respiratory and cardiac failure have received considerable attention, but very little attention has been given to mechanical ventilation during ECMO. Mechanical ventilation settings in non-ECMO studies have been shown to have an effect on survival and may also have contributed to a treatment effect in ECMO trials. Protective lung ventilation strategies established for non-ECMO-supported respiratory failure patients may not be optimal for more severe forms of respiratory failure requiring ECMO support. The influence of positive end-expiratory pressure on the reduction of the left ventricular compliance may be a matter of concern for patients receiving ECMO support for cardiac failure. The objectives of this review were to describe potential mechanisms for lung injury during ECMO for respiratory or cardiac failure, to assess the possible benefits from the use of ultra-protective lung ventilation strategies and to review published guidelines and expert opinions available on mechanical ventilation-specific management of patients requiring ECMO, including mode and ventilator settings. Articles were identified through a detailed search of PubMed, Ovid, Cochrane databases and Google Scholar. Additional references were retrieved from the selected studies. Growing evidence suggests that mechanical ventilation settings are important in ECMO patients to minimize further lung damage and improve outcomes. An ultra-protective ventilation strategy may be optimal for mechanical ventilation during ECMO for respiratory failure. The effects of airway pressure on right and left ventricular afterload should be considered during venoarterial ECMO support of cardiac failure. Future studies are needed to better understand the potential impact of invasive mechanical ventilation modes and settings on outcomes.

## Review

## Introduction

Over the past decade, the use of two distinct modalities of extracorporeal membrane oxygenation (ECMO) for respiratory and cardiac support in adults has increased. Venovenous (VV)-ECMO may be initiated as a treatment strategy for patients with severe acute respiratory failure, including adult respiratory distress syndrome (ARDS) [1-5], as a salvage therapy for patients with profound gas-exchange abnormalities despite positive-pressure ventilation. Additionally, partial extracorporeal support systems have been suggested for less severe respiratory failure as an adjunct to invasive mechanical ventilation (MV) for patients who have excessively high inspiratory airway pressures or who are unable to tolerate volume-limited and pressure-limited strategies. These devices predominately remove carbon dioxide (CO_2_) from the blood and provide limited oxygenation [6-8]. Such systems are often classified as extracorporeal carbon dioxide removal (ECCO_2_R) systems and cannot provide complete respiratory support. VV-ECMO and ECCO_2_R may now be considered management options for chronic end-stage respiratory failure where MV is contraindicated or undesirable; for example, as a bridge to lung transplantation in patients with cystic fibrosis who need to perform airway clearance techniques for sputum retention [9,10]. ECCO_2_R has also been described for chronic obstructive pulmonary disease patients with prolonged weaning of invasive MV [11]. Venoarterial (VA)-ECMO is a rapidly deployable treatment option for temporary circulatory assistance in patients with cardiogenic shock or refractory cardiac arrest [12-14] secondary to a large number of acute and chronic cardiac illnesses.

MV management during VV-ECMO and VA-ECMO has received scant attention to date despite high-level evidence to support low-tidal-volume ventilation strategies to improve survival [15,16]. The design of randomized controlled trials of ECMO in ARDS did not use standardized protective ventilation in the interventional arm [8,17] or in the control arm [3], which could have jeopardized the success of the ECMO treatment in these trials. MV settings may have important implications in both modes of ECMO (that is, VV-ECMO and VA-ECMO). Patients with the most severe forms of lung injury are likely to be particularly susceptible to ventilator-associated lung injury. Limiting stress and strain with a volume-limited and pressure-limited protective ventilation strategy beyond that recommended for patients with ARDS could provide additional benefit during ECMO support [4,18,19]. For patients with severe cardiac failure supported with VA-ECMO, pulmonary artery blood flow may be severely reduced and the maintenance of normal alveolar ventilation might lead to severe over-ventilation of the lungs [20]. Positive airway pressure settings will also affect right and left ventricular load in both VV-ECMO and VA-ECMO [21].

Brief guidelines for the use of ECMO [22] and expert points of views [3,23] have been published, mostly during the recent influenza A(H1N1) pandemic [24]. These publications are based on clinician preference, experience of centers with high case volumes, previous randomized trials [3] and local resource availability.

While there are extensive reviews on ECMO management [23,25-29], there is a significant knowledge gap in understanding the benefits and risks of MV during ECMO. Unlike previous reviews on ECMO [23,27,29], this review will focus on MV during ECMO. The purpose is to highlight the interactions between MV, ECMO and the pathophysiology of severe acute respiratory and cardiac failure. A second purpose is to provide evidence of the risks associated with MV during ECMO. Additionally, this review will summarize current guidelines, describe new strategies advocated for MV, provide evidence-based criteria that can be used for MV during ECMO and discuss what future studies are needed to address the evidence gap in this area.

## Physiological considerations and possible mechanisms for harm and benefit of mechanical ventilation during venovenous extracorporeal membrane oxygenation

### Nonpulmonary gas exchange: how much gas exchange can extracorporeal membrane oxygenation provide?

The extent of nonpulmonary gas exchange required during ECMO is directly related to the limitation of pulmonary gas exchange. The amount of oxygen supplied to the patient by the ECMO circuit is limited by the maximal oxygen delivery of the membrane (that is, membrane outlet–inlet oxygen content). The current generation of ECMO membranes can deliver up to 450 ml oxygen/minute [30]. Actual patient oxygen delivery from an ECMO circuit is affected by the rate of circuit blood flow, the hemoglobin concentration and the oxyhemoglobin saturation of the venous blood (partly reflecting the level of recirculation). Of note, with VV-ECMO the circuit blood flow is related to both the inflow cannula diameter and the cardiac output [31]. The CO_2_ content in blood is higher than the oxygen content, and is rapidly diffusible. CO_2_ transfer provided by current membranes may exceed 450 ml/minute depending on the ratio of gas to blood flow in the membrane and the CO_2_ partial pressure. Higher sweep gas flow and higher CO_2_ partial pressure in the oxygenator blood result in greater CO_2_ clearance. CO_2_ removal is therefore easily controlled with sweep gas flow settings [32].

### Minimizing ventilator-induced lung injury

MV can activate inflammation and worsen the pulmonary damage of the underlying disease, leading to ventilator-induced lung injury (VILI) [33]. Three possible causal mechanisms of VILI may be modifiable with the use of ECMO.

First is the alveolar strain, which represents the amount of aerated lung receiving ventilation [34,35]. In 2000, the ARDS Network published a multicenter randomized clinical trial where a strategy aimed at maintaining plateau pressure ≤30 cmH_2_O with an initial tidal volume of ≈ 6 ml/kg predicted body weight (PBW) was compared with traditional ventilation treatment that involved an initial tidal volume of ≈ 12 ml/kg PBW [15]. The protective ventilation, which minimizes the alveolar strain physiological concept, was associated with a decreased mortality of 22%. Patients at many centers who have received ECMO for severe ARDS have a very low arterial partial pressure of oxygen/fraction of inspired oxygen ratio (≈50 mmHg) [1,4] and a very high acute injury score [1,4]. In addition, these patients have a very small area of normally aerated alveoli located in the nondependent lung, a large consolidated or nonaerated region located in the dependent lung along the vertical axis [36-38] and frequent infiltration of all of the four lung’s quadrants on chest radiographs [1]. As the aerated compartment receives the largest part of the tidal volume [37,39], these severely unwell patients with a large amount of collapsed lung may be exposed to VILI despite low-tidal-volume ventilation strategies [40]. Limitation of the alveolar strain is a major concern of patients with ARDS receiving MV during ECMO.

A second mechanism of VILI is due to repeated intra-tidal alveolar opening and closing (atelectrauma), defined as the amount of collapsed lung tissue that is re-opened during inspiration and re-collapsed during expiration [41-43]. The challenge is to find the right ventilator settings to avoid intra-tidal alveolar opening and closing while limiting the risk of alveolar overdistension or strain [44]. Combining a low tidal volume with high levels of positive end-expiratory pressures (PEEP) appears to be important. Caironi and colleagues showed similar alveolar strain after application of 15 cmH_2_O PEEP in two distinct groups of 34 ARDS/acute lung injury patients (that is, higher vs. lower percentage of potentially recruitable lung groups) [41], suggesting that the beneficial impact of reducing intra-tidal alveolar opening and closing by increasing PEEP prevailed over the effects of increasing alveolar strain. Of note, despite improving oxygenation [45,46] and reducing the duration of MV [46], a strategy for setting PEEP aimed at increasing alveolar recruitment while limiting hyperinflation did not significantly reduce mortality in ARDS [45-47].

Finally, oxygen lung toxicity from a high fraction of inspired oxygen in lung areas with a low ventilation–perfusion ratio might alone cause reabsorption atelectasis [48-51]. Such areas are frequent in ARDS, and Aboab and colleagues showed in mechanically ventilated patients with acute lung injury that the breathing of pure oxygen leads to derecruitment, which is prevented by high PEEP [52]. The challenge of MV settings with ECMO, particularly when lung function is severely impaired, is to minimize these pitfalls.

## Physiological considerations and possible mechanisms for harm and benefit of mechanical ventilation during venoarterial extracorporeal membrane oxygenation

Patients with cardiac failure receiving VA-ECMO often have abnormal lung function that may be associated with ARDS. Considerations from the previous section may also apply to this group. However, the major cardiovascular effect associated with PEEP is reduction in cardiac output. Although the effect of PEEP on cardiac output is complex, the decrease is caused predominantly by decreasing the right ventricular preload and direct heart–lung interaction [53]. By increasing the intrathoracic pressure, PEEP can increase pulmonary vascular resistance, which may cause right ventricular overload and reduced left ventricular compliance. Patients who have received VA-ECMO with predominately right ventricular failure can be adversely affected by high PEEP [54,55]. Conversely, patients with predominately left ventricular failure supported with VA-ECMO may develop pulmonary edema despite adequate systemic support and often benefit from the application of high PEEP [34].

Additionally, VA-ECMO may dramatically reduce pulmonary blood flow as a result of pulmonary shunting. If normal lung ventilation is maintained in this setting, severe local alkalosis might result. To date, this potential deleterious effect has not been widely described and clinical consequences are still unknown. However, some authors have suggested that decreased lung perfusion with VA-ECMO may accelerate pulmonary vascular thrombosis in the presence of severe lung injury [17,20].

## Evidence and current recommendations

To date, animal data, observational studies and previous randomized trials may give a physiologic rationale to promote ultra-protective ventilation during ECMO.

### Mechanical ventilation settings: tidal volume and plateau pressure limitation

**Figure 1:**
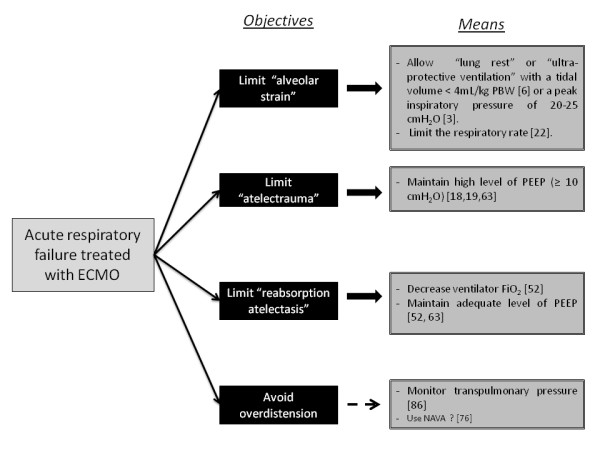
**Specifications of mechanical ventilation with extracorporeal membrane oxygenation for patients with severe acute respiratory failure.** ECMO, extracorporeal membrane oxygenation; FiO_2_, fraction of inspired oxygen; NAVA, neurally adjusted ventilator assist; PBW, predicted body weight; PEEP, positive end-expiratory pressure.

The main objectives of MV during ECMO for patients with severe acute respiratory failure are summarized in Figure 1. However, multiple approaches to ventilation could be acceptable [29]. By directly removing CO_2_ from the blood, ECMO enables lung-protective ventilation. Without ECMO, difficulty maintaining adequate alveolar ventilation is one limitation to the use of a protective ventilation strategy – exposing patients to potential side effects of subsequent hypercapnia, such as intracranial pressure elevation, pulmonary hypertension, depressed myocardial contractility and a reduction in renal blood flow [56,57].

Using tidal volume <4 ml/kg PBW is thus recommended with ECMO [29] and is often referred to imprecisely as lung rest or ultra-protective ventilation [3,7]. The concept of ultra-protective MV was suggested and investigated in animal studies [58]. Using a rat model of acid-induced lung injury, Frank and colleagues showed that a tidal volume reduction from 12 to 6 to 3 ml/kg, with the same level of PEEP (10 cmH_2_O), decreased pulmonary edema and lung injury, and increased protection of the alveolar epithelium [58]. In addition, *post-hoc* analysis of the ARDS Network data in a multivariable logistic regression model showed that lower tidal volume assignment and lower plateau pressure quartile were significant predictors of lower mortality [59]. Favorable outcome of patients with ARDS treated with a strong tidal volume reduction until 1.9 ml/kg PBW with ECMO [19] and with ECCO_2_R [18] have been reported. Terragni and colleagues used a system that predominately removed CO_2_ to reduce tidal volume <6 ml/kg PBW and observed a reduction of pulmonary cytokine concentration [60]. However, survival benefit from ultra-protective lung ventilation was not seen in a recent multicenter randomized controlled trial [6]. Bein and colleagues compared a very low tidal volume ventilation strategy (≈ 3 ml/kg PBW) combined with ECCO_2_R to lower tidal ventilation (≈ 6 ml/kg PBW) without the extracorporeal device in 79 patients with ARDS. The number of ventilator-free days at day 60 and the mortality rates were not significantly different between the two study groups. However, promising results were found in patients with severe hypoxemia with a lower number of ventilator-free days at 60 days in the control group [6]. In addition, Pham and colleagues recently showed, in a cohort of 123 patients with influenza A(H1N1)-induced ARDS, that a higher plateau pressure on the first day of VV-ECMO for acute respiratory failure was significantly associated with ICU death (odds ratio = 1.33, 95% confidence interval = 1.14 to 1.59, *P* <0.01) [4]. It is worth noting that the ultra-protective ventilation strategy was associated with the use of high PEEP levels in all cases [6,18,60].

Using a pressure control approach with tight limitation of the peak inspiratory pressure between 20 and 25 cmH_2_O has been suggested to be beneficial [3]. Depending on the severity of the lung disease, ultra-protective ventilation with plateau pressure limitation may lead to apneic ventilation (that is, no tidal volume) for several days. This may be particularly evident with pediatric patients [61,62], and in some cases the reduction of the tidal volume to achieve limited ventilation pressure strategy is considered so important that the result is insufficient ventilation to maintain adequate oxygen delivery. In this case, a third venous ECMO cannula to increase ECMO blood flow may be utilized rather than increasing the inspiratory pressure, which may negate the beneficial effect of the ECMO. Despite its feasibility reported on animal studies [63], pediatric studies [64] and in our daily adult practice, the long-term effect of apneic ventilation is unknown. A very low tidal volume results in dead space ventilation only. In our opinion, this must be combined with a high level of PEEP, to maintain convective ventilation for the elimination of alveolar nitrogen [63] and avoid alveolar collapse.

Although there are no large randomized studies focused on MV settings during ECMO in severe acute respiratory failure, it is reasonable, at this time, to advise an ultra-protective ventilation strategy with ECMO, based on a tidal volume reduction (that is, <4 ml/kg PBW) and on a plateau pressure reduction (that is, ≤25 cmH_2_O), provided lung recruitment with PEEP is sufficient.

For patients without ARDS treated by VA-ECMO, lung function is often abnormal. Cardiogenic pulmonary edema, postoperative lung damage and thoracic compliance reduction are frequently present after cardiac surgery, and these patients are at risk of developing ARDS. Targeting lower tidal volumes (6 to 8 ml/kg PBW) appears to decrease the incidence of adverse outcome, even without ARDS [65], and would appear to be reasonable in this population [22], as would a reduction in the respiratory rate during periods of minimal pulmonary artery blood flow. In addition, CO_2_ removal by VA-ECMO might allow a better tolerance of low tidal volume with less discomfort and dyspnea, and therefore less sedation – but this is an area for further research.

### Mechanical ventilation settings: positive end-expiratory pressure

It is important to be aware that, despite the use of ECMO, decreasing tidal volume <4 ml/kg PBW may increase atelectasis and result in severe ventilation/perfusion mismatch unless PEEP is appropriately increased [66]. Higher PEEP levels are essential [18,19,63] – probably higher than suggested by the Extracorporeal Life Support Organization (ELSO) guidelines, which suggest a modest PEEP of 10 cmH_2_O [22] – while taking into account the risk of alveolar overdistension and increased alveolar strain [44]. In addition, higher PEEP levels, can also maintain convective ventilation for the elimination of alveolar nitrogen accumulated during apneic oxygenation with ECMO [63].

High PEEP levels may have an adverse effect on hemodynamics when the patient is managed in the VV-ECMO mode by inhibiting venous return [22]. Although heart function is partially or completely replaced by VA-ECMO, high PEEP levels might also exacerbate right ventricular dysfunction and delay heart recovery. Particular caution and frequent cardiac echography monitoring should thus be advised for patients with ARDS and moderate right failure treated with VV-ECMO.

### Mechanical ventilation settings: fraction of inspired oxygen, respiratory rate

To limit pulmonary oxygen toxicity [52], the ventilator fraction of inspired oxygen should also be reduced to the minimal value to keep arterial saturation >85% [23]. Settings for the respiratory rate are debated, with some authors suggesting that rapid respiratory rates may increase mechanical lung stress [22]. Current expert opinions vary, and a broad range has been suggested from 4 to 30 cycles/minute (Table 1). In our opinion, the respiratory rate must be set to maintain pH and arterial CO_2_ partial pressure within normal ranges. A first approach could thus be to tailor the respiratory rate to the tidal volume and ECMO gas flow settings.

**Table 1:** Actual experts’ opinion regarding mechanical ventilation management with extracorporeal membrane oxygenation

**Source**	**Mechanical ventilation settings**	**Notes**
**ECMO for severe ARDS**		
ELSO guidelines [22]	Reasonable initial ventilator settings during ECMO could be:	‘These guidelines describe useful and safe practice, but these are not necessarily consensus recommendations. These guidelines are not intended as a standard of care …’
	• decelerating flow (pressure control)	Once patients stabilize and sedation can be lightened, spontaneous ventilation with pressure support ventilation can be considered
	• modest PEEP (for example, 10 cmH_2_O)	
	• low inflation pressure (for example, 10 cmH_2_O above PEEP)	
	• respiratory frequency 4 to 5 breaths per minute	
European Network of Mechanical Ventilation (REVA) [24]	Volume assist control mode with:	These recommendations were done specifically for patients with H1N1 influenza-induced ARDS
	• PEEP ≥10 cmH_2_O	
	• tidal volume reduced to obtain plateau pressure ≤20 to 25 cmH_2_O	
	• respiratory rate 6 to 20 cycles/minute	
	• FiO_2_ between 30 and 50%	
CESAR trial [3]	Lung rest settings with:	
	• peak inspiratory pressure 20 to 25 cmH_2_O	
	• PEEP between 10 and 15 cmH_2_O	
	• respiratory rate 10 cycles/minute	
	• FiO_2_ 30%	
EOLIA trial [72]	Assisted control mode with:	Multicenter, international, randomized, open trial that will evaluate the impact on the morbidity and mortality of ECMO, early instituted after the diagnosis of ARDS with an unfavorable outcome after 3 to 6 hours despite optimal ventilatory management and maximum medical treatment. The trial is still in progress
	• PEEP ≥10 cmH_2_O	
	• tidal volume reduced to obtain plateau pressure ≤20 cmH_2_O	
	• respiratory rate 10 to 30 cycles/minute	
	• or APRV with:	
	• high pressure ≤20 cmH_2_O	
	• PEEP ≥10 cmH_2_O	
**ECMO for cardiac failure (VA-ECMO)**		
ELSO guidelines [22]	‘Whether the patient is on either venovenous or venoarterial mode, the ventilator should be managed at low settings to allow lung rest’	

*APRV* airway pressure release ventilation, *ARDS* adult respiratory distress syndrome, *ELSO* Extracorporeal Life Support Organization, *ECMO* extracorporeal membrane oxygenation, *FiO*_*2*_ Fraction of inspired oxygen, *PEEP* positive end-expiratory pressure, *VA-ECMO* venoarterial extracorporeal membrane oxygenation.

### Mechanical ventilation settings: mode of mechanical ventilation

To date no study has compared different modes of MV during ECMO. The choice of mode used during ECMO must thus be guided by both physician usage pattern and local resource availability. However, an ultra-protective strategy should be advocated (Table 1).

The assist-controlled mode in pressure or volume seems to be commonly used during the initial phase of ARDS with ECMO, when patients are often deeply sedated and paralyzed and when alterations of lung compliance are greatest. With the target settings discussed previously, the pressure-controlled mode appears to be advocated, and is probably the most popular mode in the initial phase of ARDS with ECMO [3,22]. While minimizing the potential for VILI with ultra-protective ventilation, the pressure-controlled mode allows daily monitoring of the tidal volume increase as the patient’s condition improves. As the lung compliance improves, it is possible to see the gradual increase in tidal volume from very negligible tidal volume (50 ml) to 6 ml/kg PBW (inferior limit for ECMO weaning) [22]. However, while pressure control ventilation is advocated, some authors have recommended including spontaneous breathing to allow diaphragm contraction. Spontaneous breathing in any phase of the mechanical ventilator cycle is possible during airway pressure release ventilation, a ventilation mode that periodically switches between two levels of continuous positive airway pressure [67]. Combined with spontaneous breathing, airway pressure release ventilation appeared to augment the distribution of ventilation to dependent lung regions [68], to decrease the workload on the respiratory muscle [69] and to increase systemic blood flow in patients with severe ARDS [70]. As a result, airway pressure release ventilation might be an alternative mode to conventional pressure-controlled MV in ARDS with ECMO [71,72].

Prolonged controlled ventilation without diaphragmatic contraction may result in severe atrophy and increased duration of ventilatory support [73]. The pressure-assisted mode with spontaneous diaphragm contraction should therefore be used as soon as possible. Recent case reports have noticed the successful combination of neurally adjusted ventilatory assist and ECMO in patients with severely impaired lung function in the recovery phase [74,75]. The automated protective ventilation permitted with this closed-loop ventilation mode [76] may improve patient–ventilator synchrony, particularly in patients with ARDS [75].

In the case of patients with hypercapnic respiratory failure due to chronic obstructive pulmonary disease, or chronic end-stage respiratory failure treated with VV-ECMO or ECCO_2_R, the findings of recent pilot studies have suggested that the optimal management is to wean from MV as soon as possible once ECMO has been established [9,11,77]. In this case, ECMO without invasive MV, named awake ECMO, seems feasible, is relevant and has been associated with good results [9,11,77].

### Associated measures

To obtain high-flow ECMO sufficient to perform ultra-protective ventilation, ECMO cannulas with high diameter are essential [31]. Similarly, diuresis to dry weight [78] and restrictive transfusion strategies [31] should be attempted early in the patient’s management, despite volume expansion that may be needed at the initial phase of the disease. Every effort should then be made to achieve net negative fluid balance. In addition, tracheotomy is frequently done safely under ECMO in this population exposed to prolonged MV. Sedation and analgesia should be titrated as low as possible to conciliate protective ventilation with comfort and tolerance of the cannula.

### Recommendations

Many retrospective studies of patients with ARDS mechanically ventilated during ECMO did not provide details of MV settings [1,2,79]. Table 1 summarizes the views of experts in the field in the form of the ELSO guidelines [22], MV network [24] and protocols from previous [3] or ongoing [72] prospective randomized trials. For brief MV management, ELSO recommends ‘reasonable initial ventilator settings during extracorporeal life support (ECLS) … with a respiratory frequency of 4 to 5 per minute, modest PEEP (e.g. 10 cm H_2_O), and low inflation pressure (e.g. 10 cm H_2_O above PEEP, or a peak inspiratory pressure (PIP) of 20 cm H_2_O. Once patients are stabilized and sedation can be lightened, spontaneous ventilation with pressure support ventilation can be considered’ [22].

Despite current use of heterogeneous modes of MV, an ultra-protective ventilation strategy for ECMO with acute respiratory failure is commonly suggested as best practice (Table 1). To what extent we should reduce both the tidal volume and the plateau pressure to allow lung rest remains unknown and is an area for future research. Similarly, the impact of PEEP and tidal volume on the timing of recovery of heart function in patients supported with VA-ECMO is unclear and is another important area of future research.

## Evidence gap and future directions

Recent publications have suggested several directions forward.

### No future for invasive mechanical ventilation with ECMO?

In the future, will we still need invasive MV with ECMO at all? As described previously, invasive MV is a potential cause of VILI and ventilator-associated pneumonia, which can further enhance the initial lung damage. Numerous centers have reported the strategy of employing ECMO as a bridge to lung transplantation [9,80,81] without invasive ventilation.

A recent pilot study has suggested that ECMO might be used for hypercapnic respiratory failure in chronic obstructive pulmonary disease patients as an alternative to non-invasive ventilation or in the case of non-invasive ventilation failure [11]. In a proof-of-concept study, 26 patients awaiting lung transplantation who developed end-stage respiratory failure and were supported with ECMO while awake (that is, no invasive ventilation) were retrospectively compared with a historical control group [9]. The control group was supported with invasive MV as a bridge to lung transplantation. Despite the same duration of assistance, the 6-month survival after transplantation was higher in the ECMO group. The main benefits of awake ECMO were avoiding the complications of prolonged intubation, MV and sedation, as well as maintaining active physical activity while receiving ECMO, which may have improved physical fitness prior to transplantation. The same strategy was also described as feasible and safe with VA-ECMO [82]. This is briefly suggested by the ELSO guidelines as: ‘… An alternative is to extubate the patient and allow spontaneous breathing with the patient awake’ [22].

Recent advances in ECMO technology and a better understanding of the respiratory drive, in particular the source of dyspnea and discomfort, might allow the use of ECMO as an alternative to invasive MV in selected patients with ARDS [83]. Hoeper and colleagues recently reported the feasibility of VV-ECMO in six awake, non-intubated, spontaneously breathing patients with ARDS [84]. Avoiding mechanical ventilation might be of particular interest in specific patient populations that are placed at high risk with invasive ventilation; for example, patients with immunosuppression or end-stage chronic lung disease [9,11].

### Trials focus on mechanical ventilation strategies with extracorporeal membrane oxygenation

Description of actual MV management with ECMO worldwide is warranted to give the basis to design future interventional trials. For instance, evaluation of the impact of an ultra-protective lung ventilation strategy with ECMO for ARDS by a large international randomized trial is now needed.

### Monitoring mechanical ventilation settings prior to and during extracorporeal membrane oxygenation

As blood gas is a mix up of oxygen delivered both from the ECMO and from the native lung, monitoring the native lung during ECMO is very scanty. However, simple beside tools are available. Daily plateau pressure and compliance monitoring is a first, but imperfect, way to monitor native lung function. In addition, if a pressure mode is used, the daily monitoring of the tidal volume obtained may be valuable information. Although there is no published evidence, continuous measurement of end-tidal CO_2_ is used by some teams to monitor native lung improvement during the ECMO course. Moreover, MV during ECMO may lead to overdistension and under-recruitment depending on the degree of heterogeneity of regional compliance [44].

Assessment of regional lung mechanics is not easy with extremely severe ARDS prior to or during ECMO. Therefore, for technical and safety reasons, thoracic computed tomography could not be routinely recommended for these patients. Electrical impedance tomography could be considered as a MV monitoring tool prior and during ECMO for ARDS. Some authors have suggested that it could be a bedside tool to identify patients in whom lung protection and reversal of hypoxemia is not achievable with MV (that is, ECMO recipient), or to identify patients who would benefit from MV with lung recruitment maneuvers (that is, ECMO nonrecipient) [85]. Similarly, Grasso and colleagues suggested the use of transpulmonary pressure (assessed by esophageal balloon) as a bedside tool to assess physiological titration of PEEP [86]. Indeed, the authors demonstrated that 50% of patients with influenza A(H1N1)-associated ARDS referred to their unit for ECMO did not receive ECMO, because the transpulmonary pressure was lower than airway pressure alone. This unexpected lower value, mainly due to low chest wall compliance, allowed a safe increase in the PEEP level, which in turn increased oxygenation and avoided the use of ECMO. This individually tailored approach could be the future direction of research to select the ‘best’ candidates for ECMO, if used as a rescue therapy for severe acute hypoxemic respiratory failure.

## Conclusions

Although the positive impact of a protective lung ventilation strategy on survival in ARDS has been clearly demonstrated, in patients receiving MV during ECMO there is limited evidence to guide practice. Based on actual and past randomized trials of ARDS with ECMO, an ultra-protective ventilation strategy that limits tidal volume to <4 ml/kg PBW, targets a very low plateau pressure (<25 cmH_2_O) and is supported by increased alveolar recruitment with PEEP may be the best option for clinicians managing these critically ill patients. As the use of ECMO increases internationally, future studies are urgently required to determine the best practice of MV during ECMO and its impact on patient-centered outcomes.

## Abbreviations

ARDS: Adult respiratory distress syndrome; CO2: Carbon dioxide; ECCO2R: Extracorporeal carbon dioxide removal; ECMO: Extracorporeal membrane oxygenation; ELSO: Extracorporeal Life Support Organization; MV: Mechanical ventilation; PBW: Predicted body weight; PEEP: Positive end-expiratory pressure; VA-ECMO: Venoarterial extracorporeal membrane oxygenation; VILI: Ventilator-induced lung injury; VV-ECMO: Venovenous extracorporeal membrane oxygenation.

## Competing interests

AC is the primary investigator of the EOLIA trial, NCT01470703, a randomized trial of VV-ECMO supported in part by MAQUET. AC has received honoraria for lectures from MAQUET. The remaining authors declare that they have no competing interests.

## References

[B1] Davies A Jones D Bailey M Beca J Bellomo R Blackwell N Forrest P Gattas D Granger E Herkes R Jackson A McGuinness S Nair P Pellegrino V Pettila V Plunkett B Pye R Torzillo P Webb S Wilson M Ziegenfuss M Extracorporeal membrane oxygenation for 2009 influenza A(H1N1) acute respiratory distress syndrome JAMA 2009 302 1888 1895 19822628 10.1001/jama.2009.1535

[B2] Noah MA Peek GJ Finney SJ Griffiths MJ Harrison DA Grieve R Sadique MZ Sekhon JS McAuley DF Firmin RK Harvey C Cordingley JJ Price S Vuylsteke A Jenkins DP Noble DW Bloomfield R Walsh TS Perkins GD Menon D Taylor BL Rowan KM Referral to an extracorporeal membrane oxygenation center and mortality among patients with severe 2009 influenza A(H1N1) JAMA 2011 306 1659 1668 10.1001/jama.2011.1471 21976615

[B3] Peek GJ Mugford M Tiruvoipati R Wilson A Allen E Thalanany MM Hibbert CL Truesdale A Clemens F Cooper N Firmin RK Elbourne D Efficacy and economic assessment of conventional ventilatory support versus extracorporeal membrane oxygenation for severe adult respiratory failure (CESAR): a multicentre randomised controlled trial Lancet 2009 374 1351 1363 10.1016/S0140-6736(09)61069-2 19762075

[B4] Pham T Combes A Roze H Chevret S Mercat A Roch A Mourvillier B Ara-Somohano C Bastien O Zogheib E Clavel M Constan A Marie Richard JC Brun-Buisson C Brochard L Extracorporeal membrane oxygenation for pandemic influenza A(H1N1)-induced acute respiratory distress syndrome: a cohort study and propensity-matched analysis Am J Respir Crit Care Med 2013 187 276 285 10.1164/rccm.201205-0815OC 23155145

[B5] Schmidt M Zogheib E Roze H Repesse X Lebreton G Luyt CE Trouillet JL Brechot N Nieszkowska A Dupont H Ouattara A Leprince P Chastre J Combes A The PRESERVE mortality risk score and analysis of long-term outcomes after extracorporeal membrane oxygenation for severe acute respiratory distress syndrome Intensive Care Med 2013 39 1704 1713 10.1007/s00134-013-3037-2 23907497 PMC7094902

[B6] Bein T Weber-Carstens S Goldmann A Muller T Staudinger T Brederlau J Muellenbach R Dembinski R Graf BM Wewalka M Philipp A Wernecke KD Lubnow M Slutsky AS Lower tidal volume strategy (approximately 3 ml/kg) combined with extracorporeal CO(2) removal versus ‘conventional’ protective ventilation (6 ml/kg) in severe ARDS: the prospective randomized Xtravent-study Intensive Care Med 2013 39 847 856 10.1007/s00134-012-2787-6 23306584 PMC3625408

[B7] Gattinoni L Pesenti A Mascheroni D Marcolin R Fumagalli R Rossi F Iapichino G Romagnoli G Uziel L Agostoni A Kolobow T Damia G Low-frequency positive-pressure ventilation with extracorporeal CO2 removal in severe acute respiratory failure JAMA 1986 256 881 886 10.1001/jama.1986.03380070087025 3090285

[B8] Morris AH Wallace CJ Menlove RL Clemmer TP Orme JF Jr Weaver LK Dean NC Thomas F East TD Pace NL Suchyta MR Beck E Bombino M Sittig DF Bohm S Hoffmann B Becks H Butler S Pearl J Rasmusson B Randomized clinical trial of pressure-controlled inverse ratio ventilation and extracorporeal CO2 removal for adult respiratory distress syndrome Am J Respir Crit Care Med 1994 149 295 305 10.1164/ajrccm.149.2.8306022 8306022

[B9] Fuehner T Kuehn C Hadem J Wiesner O Gottlieb J Tudorache I Olsson KM Greer M Sommer W Welte T Haverich A Hoeper MM Warnecke G Extracorporeal membrane oxygenation in awake patients as bridge to lung transplantation Am J Respir Crit Care Med 2012 185 763 768 10.1164/rccm.201109-1599OC 22268135

[B10] Ricci D Boffini M Del Sorbo L El Qarra S Comoglio C Ribezzo M Bonato R Ranieri VM Rinaldi M The use of CO2 removal devices in patients awaiting lung transplantation: an initial experience Transplant Proc 2010 42 1255 1258 10.1016/j.transproceed.2010.03.117 20534274

[B11] Burki NK Mani RK Herth FJ Schmidt W Teschler H Bonin F Becker H Randerath WJ Stieglitz S Hagmeyer L Priegnitz C Pfeifer M Blaas SH Putensen C Theuerkauf N Quintel M Moerer O A novel extracorporeal CO(2) removal system: results of a pilot study of hypercapnic respiratory failure in patients with COPD Chest 2013 143 678 686 23460154 10.1378/chest.12-0228 PMC3590884

[B12] Chen YS Chao A Yu HY Ko WJ Wu IH Chen RJ Huang SC Lin FY Wang SS Analysis and results of prolonged resuscitation in cardiac arrest patients rescued by extracorporeal membrane oxygenation J Am Coll Cardiol 2003 41 197 203 12535808 10.1016/s0735-1097(02)02716-x

[B13] Magovern GJ Jr Simpson KA Extracorporeal membrane oxygenation for adult cardiac support: the Allegheny experience Ann Thorac Surg 1999 68 655 661 10.1016/S0003-4975(99)00581-0 10475466

[B14] Smedira NG Moazami N Golding CM McCarthy PM Apperson-Hansen C Blackstone EH Cosgrove DM 3rd Clinical experience with 202 adults receiving extracorporeal membrane oxygenation for cardiac failure: survival at five years J Thorac Cardiovasc Surg 2001 122 92 102 10.1067/mtc.2001.114351 11436041

[B15] The Acute Respiratory Distress Syndrome Network Ventilation with lower tidal volumes as compared with traditional tidal volumes for acute lung injury and the acute respiratory distress syndrome. The Acute Respiratory Distress Syndrome Network N Engl J Med 2000 342 1301 1308 10793162 10.1056/NEJM200005043421801

[B16] Burns KE Adhikari NK Slutsky AS Guyatt GH Villar J Zhang H Zhou Q Cook DJ Stewart TE Meade MO Pressure and volume limited ventilation for the ventilatory management of patients with acute lung injury: a systematic review and meta-analysis PLoS One 2011 6 e14623 10.1371/journal.pone.0014623 21298026 PMC3030554

[B17] Zapol WM Snider MT Hill JD Fallat RJ Bartlett RH Edmunds LH Morris AH Peirce EC 2nd Thomas AN Proctor HJ Drinker PA Pratt PC Bagniewski A Miller RG Jr Extracorporeal membrane oxygenation in severe acute respiratory failure. A randomized prospective study JAMA 1979 242 2193 2196 10.1001/jama.1979.03300200023016 490805

[B18] Bein T Zimmermann M Hergeth K Ramming M Rupprecht L Schlitt HJ Slutsky AS Pumpless extracorporeal removal of carbon dioxide combined with ventilation using low tidal volume and high positive end-expiratory pressure in a patient with severe acute respiratory distress syndrome Anaesthesia 2009 64 195 198 10.1111/j.1365-2044.2008.05735.x 19143699

[B19] Mauri T Foti G Zanella A Bombino M Confalonieri A Patroniti N Bellani G Pesenti A Long-term extracorporeal membrane oxygenation with minimal ventilatory support: a new paradigm for severe ARDS? Minerva Anestesiol 2012 78 385 389 21617600

[B20] Ratliff JL Hill JD Fallat RJ Parrot J Tucker HJ Complications associated with membrane lung support by venoarterial perfusion Ann Thorac Surg 1975 19 537 539 10.1016/S0003-4975(10)64429-3 1130894

[B21] Luecke T Pelosi P Clinical review: Positive end-expiratory pressure and cardiac output Crit Care 2005 9 607 621 10.1186/cc3877 16356246 PMC1414045

[B22] Extracorporeal Life Support Organization ELSO Guidelines for Cardiopulmonary Extracorporeal Life Support and Patient Specific Supplements to the ELSO General Guidelines Ann Arbor, MI [http://elso.org/]

[B23] Combes A Bacchetta M Brodie D Muller T Pellegrino V Extracorporeal membrane oxygenation for respiratory failure in adults Curr Opin Crit Care 2012 18 99 104 10.1097/MCC.0b013e32834ef412 22186218

[B24] Réseau Europeen de Recherche en Ventilation Artificielle (REVA) – Syndrome de Détresse Respiratoire Aiguë lié à la grippe A(H1N1)-2009 Recommandations pour l'assistance respiratoire [http://www.revaweb.org]

[B25] Joffe AR Lequier L Robertson CM Pediatric outcomes after extracorporeal membrane oxygenation for cardiac disease and for cardiac arrest: a review ASAIO J 2012 58 297 310 10.1097/MAT.0b013e31825a21ff 22643323

[B26] Kar B Basra SS Shah NR Loyalka P Percutaneous circulatory support in cardiogenic shock: interventional bridge to recovery Circulation 2012 125 1809 1817 10.1161/CIRCULATIONAHA.111.040220 22492948

[B27] Combes A Brechot N Luyt CE Schmidt M What is the niche for extracorporeal membrane oxygenation in severe acute respiratory distress syndrome? Curr Opin Crit Care 2012 18 527 532 10.1097/MCC.0b013e328357f090 22914430

[B28] Pitsis AA Visouli AN Mechanical assistance of the circulation during cardiogenic shock Curr Opin Crit Care 2011 17 425 438 10.1097/MCC.0b013e32834a75c1 21897218

[B29] Brodie D Bacchetta M Extracorporeal membrane oxygenation for ARDS in adults N Engl J Med 2011 365 1905 1914 10.1056/NEJMct1103720 22087681

[B30] ECMO and Life Support Systems Quadrox PLS and Rotaflow Hardware and Accessories [http://www.maquet.com/content/Cardiopulmonary/Documents/Brochures/PLS_BROCHU_MCV-BR-40000145-EN-04_1010_EN_NONUS.pdf]

[B31] Schmidt M Tachon G Devilliers C Muller G Hekimian G Brechot N Merceron S Luyt CE Trouillet JL Chastre J Leprince P Combes A Blood oxygenation and decarboxylation determinants during venovenous ECMO for respiratory failure in adults Intensive Care Med 2013 39 838 846 10.1007/s00134-012-2785-8 23291732

[B32] MacLaren G Combes A Bartlett RH Contemporary extracorporeal membrane oxygenation for adult respiratory failure: life support in the new era Intensive Care Med 2012 38 210 220 10.1007/s00134-011-2439-2 22147116

[B33] Dreyfuss D Basset G Soler P Saumon G Intermittent positive-pressure hyperventilation with high inflation pressures produces pulmonary microvascular injury in rats Am Rev Respir Dis 1985 132 880 884 3901844 10.1164/arrd.1985.132.4.880

[B34] Dreyfuss D Soler P Basset G Saumon G High inflation pressure pulmonary edema. Respective effects of high airway pressure, high tidal volume, and positive end-expiratory pressure Am Rev Respir Dis 1988 137 1159 1164 10.1164/ajrccm/137.5.1159 3057957

[B35] Gattinoni L Pesenti A The concept of ‘baby lung’ Intensive Care Med 2005 31 776 784 10.1007/s00134-005-2627-z 15812622

[B36] Gattinoni L Caironi P Cressoni M Chiumello D Ranieri VM Quintel M Russo S Patroniti N Cornejo R Bugedo G Lung recruitment in patients with the acute respiratory distress syndrome N Engl J Med 2006 354 1775 1786 10.1056/NEJMoa052052 16641394

[B37] Gattinoni L Caironi P Pelosi P Goodman LR What has computed tomography taught us about the acute respiratory distress syndrome? Am J Respir Crit Care Med 2001 164 1701 1711 10.1164/ajrccm.164.9.2103121 11719313

[B38] Rouby JJ A lung computed tomographic assessment of positive end-expiratory pressure-induced lung overdistension Am J Respir Crit Care Med 2000 161 1396 1397 10764339 10.1164/ajrccm.161.4.16147b

[B39] Vieira SR Puybasset L Lu Q Richecoeur J Cluzel P Coriat P Rouby JJ A scanographic assessment of pulmonary morphology in acute lung injury. Significance of the lower inflection point detected on the lung pressure–volume curve Am J Respir Crit Care Med 1999 159 1612 1623 10.1164/ajrccm.159.5.9805112 10228135

[B40] Terragni PP Rosboch G Tealdi A Corno E Menaldo E Davini O Gandini G Herrmann P Mascia L Quintel M Slutsky AS Gattinoni L Ranieri VM Tidal hyperinflation during low tidal volume ventilation in acute respiratory distress syndrome Am J Respir Crit Care Med 2007 175 160 166 10.1164/rccm.200607-915OC 17038660

[B41] Caironi P Cressoni M Chiumello D Ranieri M Quintel M Russo SG Cornejo R Bugedo G Carlesso E Russo R Caspani L Gattinoni L Lung opening and closing during ventilation of acute respiratory distress syndrome Am J Respir Crit Care Med 2010 181 578 586 10.1164/rccm.200905-0787OC 19910610

[B42] Mead J Takishima T Leith D Stress distribution in lungs: a model of pulmonary elasticity J Appl Physiol 1970 28 596 608 5442255 10.1152/jappl.1970.28.5.596

[B43] Muscedere JG Mullen JB Gan K Slutsky AS Tidal ventilation at low airway pressures can augment lung injury Am J Respir Crit Care Med 1994 149 1327 1334 10.1164/ajrccm.149.5.8173774 8173774

[B44] Grasso S Stripoli T Sacchi M Trerotoli P Staffieri F Franchini D De Monte V Valentini V Pugliese P Crovace A Driessen B Fiore T Inhomogeneity of lung parenchyma during the open lung strategy: a computed tomography scan study Am J Respir Crit Care Med 2009 180 415 423 10.1164/rccm.200901-0156OC 19542479

[B45] Meade MO Cook DJ Guyatt GH Slutsky AS Arabi YM Cooper DJ Davies AR Hand LE Zhou Q Thabane L Austin P Lapinsky S Baxter A Russell J Skrobik Y Ronco JJ Stewart TE Ventilation strategy using low tidal volumes, recruitment maneuvers, and high positive end-expiratory pressure for acute lung injury and acute respiratory distress syndrome: a randomized controlled trial JAMA 2008 299 637 645 10.1001/jama.299.6.637 18270352

[B46] Mercat A Richard JC Vielle B Jaber S Osman D Diehl JL Lefrant JY Prat G Richecoeur J Nieszkowska A Gervais C Baudot J Bouadma L Brochard L Positive end-expiratory pressure setting in adults with acute lung injury and acute respiratory distress syndrome: a randomized controlled trial JAMA 2008 299 646 655 10.1001/jama.299.6.646 18270353

[B47] Brower RG Lanken PN MacIntyre N Matthay MA Morris A Ancukiewicz M Schoenfeld D Thompson BT Higher versus lower positive end-expiratory pressures in patients with the acute respiratory distress syndrome N Engl J Med 2004 351 327 336 15269312 10.1056/NEJMoa032193

[B48] Dantzker DR Wagner PD West JB Proceedings: Instability of poorly ventilated lung units during oxygen breathing J Physiol 1974 242 72P 4455844

[B49] Rothen HU Sporre B Engberg G Wegenius G Reber A Hedenstierna G Prevention of atelectasis during general anaesthesia Lancet 1995 345 1387 1391 10.1016/S0140-6736(95)92595-3 7760608

[B50] Santos C Ferrer M Roca J Torres A Hernandez C Rodriguez-Roisin R Pulmonary gas exchange response to oxygen breathing in acute lung injury Am J Respir Crit Care Med 2000 161 26 31 10.1164/ajrccm.161.1.9902084 10619793

[B51] Suter PM Fairley HB Schlobohm RM Shunt, lung volume and perfusion during short periods of ventilation with oxygen Anesthesiology 1975 43 617 627 10.1097/00000542-197512000-00003 1103655

[B52] Aboab J Jonson B Kouatchet A Taille S Niklason L Brochard L Effect of inspired oxygen fraction on alveolar derecruitment in acute respiratory distress syndrome Intensive Care Med 2006 32 1979 1986 10.1007/s00134-006-0382-4 17019545

[B53] Fessler HE Heart-lung interactions: applications in the critically ill Eur Respir J 1997 10 226 237 10.1183/09031936.97.10010226 9032519

[B54] Jardin F Acute leftward septal shift by lung recruitment maneuver Intensive Care Med 2005 31 1148 1149 10.1007/s00134-005-2733-y 16096750

[B55] Jardin F Vieillard-Baron A Monitoring of right-sided heart function Curr Opin Crit Care 2005 11 271 279 10.1097/01.ccx.0000158847.56107.55 15928478

[B56] Feihl F Eckert P Brimioulle S Jacobs O Schaller MD Melot C Naeije R Permissive hypercapnia impairs pulmonary gas exchange in the acute respiratory distress syndrome Am J Respir Crit Care Med 2000 162 209 215 10.1164/ajrccm.162.1.9907119 10903243

[B57] Feihl F Perret C Permissive hypercapnia. How permissive should we be? Am J Respir Crit Care Med 1994 150 1722 1737 10.1164/ajrccm.150.6.7952641 7952641

[B58] Frank JA Gutierrez JA Jones KD Allen L Dobbs L Matthay MA Low tidal volume reduces epithelial and endothelial injury in acid-injured rat lungs Am J Respir Crit Care Med 2002 165 242 249 10.1164/ajrccm.165.2.2108087 11790662

[B59] Hager DN Krishnan JA Hayden DL Brower RG Tidal volume reduction in patients with acute lung injury when plateau pressures are not high Am J Respir Crit Care Med 2005 172 1241 1245 10.1164/rccm.200501-048CP 16081547 PMC2718413

[B60] Terragni PP Del Sorbo L Mascia L Urbino R Martin EL Birocco A Faggiano C Quintel M Gattinoni L Ranieri VM Tidal volume lower than 6 ml/kg enhances lung protection: role of extracorporeal carbon dioxide removal Anesthesiology 2009 111 826 835 10.1097/ALN.0b013e3181b764d2 19741487

[B61] Kumpers P Nickel N Lukasz A Golpon H Westerkamp V Olsson KM Jonigk D Maegel L Bockmeyer CL David S Hoeper MM Circulating angiopoietins in idiopathic pulmonary arterial hypertension Eur Heart J 2010 31 2291 2300 10.1093/eurheartj/ehq226 20601390

[B62] Zabrocki LA Brogan TV Statler KD Poss WB Rollins MD Bratton SL Extracorporeal membrane oxygenation for pediatric respiratory failure: survival and predictors of mortality Critical Care Med 2011 39 364 370 10.1097/CCM.0b013e3181fb7b35 20959787

[B63] Nielsen ND Kjaergaard B Koefoed-Nielsen J Steensen CO Larsson A Apneic oxygenation combined with extracorporeal arteriovenous carbon dioxide removal provides sufficient gas exchange in experimental lung injury ASAIO J 2008 54 401 405 10.1097/MAT.0b013e31817e2b5f 18645358

[B64] Somaschini M Bellan C Locatelli G Glauber M Colombo A Extracorporeal membrane oxygenation with veno-venous bypass and apneic oxygenation for treatment of severe neonatal respiratory failure Int J Artif Organs 1995 18 574 578 8647586

[B65] Serpa Neto A Cardoso SO Manetta JA Pereira VG Esposito DC Pasqualucci Mde O Damasceno MC Schultz MJ Association between use of lung-protective ventilation with lower tidal volumes and clinical outcomes among patients without acute respiratory distress syndrome: a meta-analysis JAMA 2012 308 1651 1659 10.1001/jama.2012.13730 23093163

[B66] Dembinski R Hochhausen N Terbeck S Uhlig S Dassow C Schneider M Schachtrupp A Henzler D Rossaint R Kuhlen R Pumpless extracorporeal lung assist for protective mechanical ventilation in experimental lung injury Crit Care Med 2007 35 2359 2366 10.1097/01.CCM.0000281857.87354.A5 17944027

[B67] Hormann C Baum M Putensen C Mutz NJ Benzer H Biphasic positive airway pressure (BIPAP) – a new mode of ventilatory support Eur J Anaesthesiol 1994 11 37 42 8143712

[B68] Yoshida T Rinka H Kaji A Yoshimoto A Arimoto H Miyaichi T Kan M The impact of spontaneous ventilation on distribution of lung aeration in patients with acute respiratory distress syndrome: airway pressure release ventilation versus pressure support ventilation Anesth Analg 2009 109 1892 1900 10.1213/ANE.0b013e3181bbd918 19923518

[B69] Hering R Zinserling J Wrigge H Varelmann D Berg A Kreyer S Putensen C Effects of spontaneous breathing during airway pressure release ventilation on respiratory work and muscle blood flow in experimental lung injury Chest 2005 128 2991 2998 10.1378/chest.128.4.2991 16236977

[B70] Putensen C Mutz NJ Putensen-Himmer G Zinserling J Spontaneous breathing during ventilatory support improves ventilation–perfusion distributions in patients with acute respiratory distress syndrome Am J Respir Crit Care Med 1999 159 1241 1248 10.1164/ajrccm.159.4.9806077 10194172

[B71] EOLIA Trial [http://www.clinicaltrials.gov/ct2/show/NCT01470703?term=EOLIA+ECMO&rank=1]

[B72] Combes A Extracorporeal membrane oxygenation (ECMO) for severe acute respiratory distress syndrome (ARDS). The EOLIA (ECMO to rescue Lung Injury in severe ARDS) trial: a multicenter, international, randomized, controlled open trial Reanimation 2011 20 49 61

[B73] Levine S Nguyen T Taylor N Friscia ME Budak MT Rothenberg P Zhu J Sachdeva R Sonnad S Kaiser LR Rubinstein NA Powers SK Shrager JB Rapid disuse atrophy of diaphragm fibers in mechanically ventilated humans N Engl J Med 2008 358 1327 1335 10.1056/NEJMoa070447 18367735

[B74] Bein T Osborn E Hofmann HS Zimmermann M Philipp A Schlitt HJ Graf BM Successful treatment of a severely injured soldier from Afghanistan with pumpless extracorporeal lung assist and neurally adjusted ventilatory support Int J Emerg Med 2010 3 177 179 10.1007/s12245-010-0192-x 21031042 PMC2926866

[B75] Mauri T Bellani G Grasselli G Confalonieri A Rona R Patroniti N Pesenti A Patient-ventilator interaction in ARDS patients with extremely low compliance undergoing ECMO: a novel approach based on diaphragm electrical activity Intensive Care Med 2013 39 282 291 10.1007/s00134-012-2755-1 23196419

[B76] Karagiannidis C Lubnow M Philipp A Riegger GA Schmid C Pfeifer M Mueller T Autoregulation of ventilation with neurally adjusted ventilatory assist on extracorporeal lung support Intensive Care Med 2010 36 2038 2044 10.1007/s00134-010-1982-6 20689930

[B77] Del Sorbo L Ranieri VM Keshavjee S Extracorporeal membrane oxygenation as ‘bridge’ to lung transplantation: what remains in order to make it standard of care? Am J Respir Crit Care Med 2012 185 699 701 10.1164/rccm.201202-0193ED 22467804

[B78] Wiedemann HP Wheeler AP Bernard GR Thompson BT Hayden D deBoisblanc B Connors AF Jr Hite RD Harabin AL Comparison of two fluid-management strategies in acute lung injury N Engl J Med 2006 354 2564 2575 16714767 10.1056/NEJMoa062200

[B79] Patroniti N Zangrillo A Pappalardo F Peris A Cianchi G Braschi A Iotti GA Arcadipane A Panarello G Ranieri VM Terragni P Antonelli M Gattinoni L Oleari F Pesenti A The Italian ECMO network experience during the 2009 influenza A(H1N1) pandemic: preparation for severe respiratory emergency outbreaks Intensive Care Med 2011 37 1447 1457 10.1007/s00134-011-2301-6 21732167 PMC7080128

[B80] Lang G Taghavi S Aigner C Renyi-Vamos F Jaksch P Augustin V Nagayama K Ghanim B Klepetko W Primary lung transplantation after bridge with extracorporeal membrane oxygenation: a plea for a shift in our paradigms for indications Transplantation 2012 93 729 736 10.1097/TP.0b013e318246f8e1 22415051

[B81] Mason DP Thuita L Nowicki ER Murthy SC Pettersson GB Blackstone EH Should lung transplantation be performed for patients on mechanical respiratory support? The US experience J Thorac Cardiovasc Surg 2010 139 765 773 e1 10.1016/j.jtcvs.2009.09.031 19931096

[B82] Olsson KM Simon A Strueber M Hadem J Wiesner O Gottlieb J Fuehner T Fischer S Warnecke G Kuhn C Haverich A Welte T Hoeper MM Extracorporeal membrane oxygenation in nonintubated patients as bridge to lung transplantation Am J Transplant 2010 10 2173 2178 10.1111/j.1600-6143.2010.03192.x 20636463

[B83] Wiesner O Hadem J Sommer W Kuhn C Welte T Hoeper MM Extracorporeal membrane oxygenation in a nonintubated patient with acute respiratory distress syndrome Eur Respir J 2012 40 1296 1298 10.1183/09031936.00076912 22878882

[B84] Hoeper MM Wiesner O Hadem J Wahl O Suhling H Duesberg C Sommer W Warnecke G Greer M Boenisch O Busch M Kielstein JT Schneider A Haverich A Welte T Kuhn C Extracorporeal membrane oxygenation instead of invasive mechanical ventilation in patients with acute respiratory distress syndrome Intensive Care Med 2013 39 2056 2057 10.1007/s00134-013-3052-3 23921981

[B85] Camporota L Smith J Barrett N Beale R Assessment of regional lung mechanics with electrical impedance tomography can determine the requirement for ECMO in patients with severe ARDS Intensive Care Med 2012 38 2086 2087 10.1007/s00134-012-2701-2 22990870

[B86] Grasso S Terragni P Birocco A Urbino R Del Sorbo L Filippini C Mascia L Pesenti A Zangrillo A Gattinoni L Ranieri VM ECMO criteria for influenza A (H1N1)-associated ARDS: role of transpulmonary pressure Intensive Care Med 2012 38 395 403 10.1007/s00134-012-2490-7 22323077

